# Outer Membrane Vesicle-Mediated Export of Processed PrtV Protease from *Vibrio cholerae*

**DOI:** 10.1371/journal.pone.0134098

**Published:** 2015-07-29

**Authors:** Pramod K. Rompikuntal, Svitlana Vdovikova, Marylise Duperthuy, Tanya L. Johnson, Monika Åhlund, Richard Lundmark, Jan Oscarsson, Maria Sandkvist, Bernt Eric Uhlin, Sun Nyunt Wai

**Affiliations:** 1 Department of Molecular Biology, Umeå University, Umeå, S-90187, Sweden; 2 The Laboratory for Molecular Infection Medicine Sweden (MIMS), Umeå University, Umeå, S-90187, Sweden; 3 Department of Microbiology and Immunology, University of Michigan Medical School, Ann Arbor, Michigan, United States of America; 4 Department of Medical Biochemistry and Biophysics, Umeå University, S-90187 Umeå, Sweden; 5 Oral Microbiology, Department of Odontology, Umeå University, S-90187 Umeå, Sweden; University of Illinois at Chicago College of Medicine, UNITED STATES

## Abstract

**Background:**

Outer membrane vesicles (OMVs) are known to release from almost all Gram-negative bacteria during normal growth. OMVs carry different biologically active toxins and enzymes into the surrounding environment. We suggest that OMVs may therefore be able to transport bacterial proteases into the target host cells. We present here an analysis of the *Vibrio cholerae* OMV-associated protease PrtV.

**Methodology/Principal Findings:**

In this study, we demonstrated that PrtV was secreted from the wild type *V*. *cholerae* strain C6706 via the type II secretion system in association with OMVs. By immunoblotting and electron microscopic analysis using immunogold labeling, the association of PrtV with OMVs was examined. We demonstrated that OMV-associated PrtV was biologically active by showing altered morphology and detachment of cells when the human ileocecum carcinoma (HCT8) cells were treated with OMVs from the wild type *V*. *cholerae* strain C6706 whereas cells treated with OMVs from the *prtV* isogenic mutant showed no morphological changes. Furthermore, OMV-associated PrtV protease showed a contribution to bacterial resistance towards the antimicrobial peptide LL-37.

**Conclusion/Significance:**

Our findings suggest that OMVs released from *V*. *cholerae* can deliver a processed, biologically active form of PrtV that contributes to bacterial interactions with target host cells.

## Introduction

*V*. *cholerae* is the causal microorganism of human diarrheal disease cholera that leads to severe loss of fluid and electrolytes. The non-capsulated O1 and the encapsulated O139 are known to cause the cholera disease among over 200 serogroups identified [1,2]. *V*. *cholerae* is a free-living natural inhabitant of estuarine and coastal waters throughout world. This bacterium can survive in conditions of varying temperature, pH, and salinity. It can also survive in the presence of bacteriovorous predators such as ciliates and flagellates [3,4]. Secreted cholera toxin (CTX) is a major virulence factor responsible for causing the cholera disease [5]. Additional secreted virulence factors have been described, including the hemagglutinin/protease (HAP), the multifunctional autoprocessing RTX toxin and hemolysin A/cytolysin (VCC) [6,7].

In order to fully understand the pathogenesis and environmental persistence of *V*. *cholerae*, it is essential to study not only the factors important for its survival inside the human host, but also factors that might be essential for its environmental adaptation. In our earlier studies, we established *Caenorhabditis elegans* as a model organism for the analysis of *V*. *cholerae* factors involved in host interactions and survival of bacteria in the environment and demonstrated that an extracellular protease, PrtV is a factor being necessary for killing *C*. *elegans* [8]. We also showed that PrtV was important for the survival of *V*. *cholerae* against the grazing by the flagellate *Cafeteria roenbergensis* and the ciliate *Tetrahymena pyriformis* [8]. PrtV causes tissue damage by directly degrading substrate proteins in host tissues, thereby inducing cell rounding and detachment of tissue culture cells [9]. In our earlier studies, we demonstrated that PrtV could modulate host inflammatory responses by interacting with *V*. *cholerae* cytolysin [10]

PrtV, a Zn^2+^-binding extracellular protease belonging to the M6 metalloprotease family, contains M6 peptidase domain harboring conserved zinc-binding motifs (HEXXH) [8,9,11]. The biological functions of C-terminal two putative polycystic kidney disease domains (PKD1 and PKD2) has not yet been fully investigated, although they have been suggested to be involved in protein-protein or protein-carbohydrate interaction [12]. Our previous studies provided a crystal structure model of the PKD1 domain from *V*. *cholerae* PrtV (residues 755–838) and revealed a Ca^++^-binding site which could control domain linker flexibility, presumably playing an important structural role by providing stability to the PrtV protein [13]. The biological roles of both PKD1 and PKD2 domains remain unknown. Although our results from the *C*. *elegans* model [8] and from human ileocecum carcinoma (HCT8) cell toxicity assays [9] support that PrtV is disseminated as a biologically active protein, the mechanism(s) for its secretion is yet unknown.

Membrane vesicles stand for a very basic and relevant mode of protein release by bacteria, which has recently been referred to as the “Type 0” (zero) secretion system [14]. Outer membrane vesicles (OMVs) (diameter ≈20–200 nm) are constantly discharged from the surface of the Gram-negative bacteria during growth, and may entrap outer membrane proteins, LPS, phospholipids, and some periplasmic components [15–17]. Recent studies showed that OMVs from commensal and pathogenic bacterial species play a fundamental role in maturation of the innate and adaptive immune systems [18–21]. Moreover, recent and earlier studies proposed that bacterial pathogens can use OMVs to deliver virulence factors into host cells at local and distal sites [16,22–27]. Little is known, however, about the specific mechanism(s) by which OMVs are formed and released from the bacterial cells, and whether particular genes control the release of OMVs. Interestingly, Premjani et al [28] showed that in Enterohemorrhagic *E*. *coli* (EHEC), the omptin outer membrane protease OmpT could influence the OMV biogenesis.

Furthermore, recent studies demonstrated roles of OMVs in antimicrobial peptide (AMP) resistance of *Escherichia coli* and in cross-resistance to AMPs such as LL-37 and polymyxin B in *V*. *cholerae* [29–31]. In this study we have investigated if biologically active PrtV is released via OMVs and tested the hypothesis that this protease plays a role in *V*. *cholerae* resistance against host AMPs.

## Results

### Identification of PrtV in OMV preparations obtained from different *V*. *cholerae* serogroups

**Fig 1:**
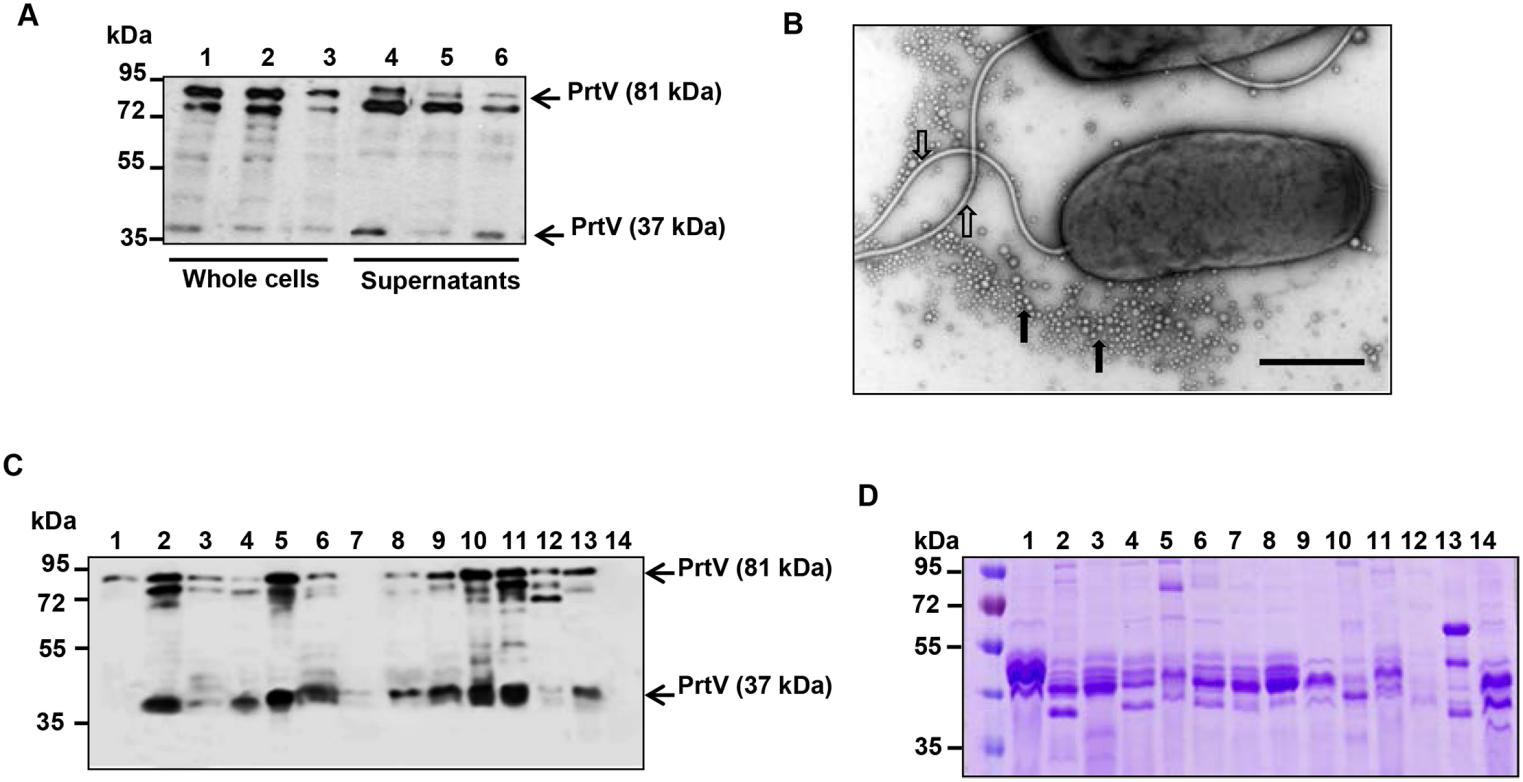
Immunoblot analyses of PrtV expression and secretion and ultrastructural analysis of *V*. *cholerae* surface structures. (A) Immunoblot analysis of expression and secretion of PrtV in different *V*. *cholerae* O1 isolates. Bacterial strains were grown at 30°C and samples were collected at OD_600_ 2.0. Samples of whole cell extracts from overnight cultures (lanes 1–3, 5 μl) and culture supernatants (lanes 4–6, 10 μl, corresponding to tenfold concentration compared with the whole cell samples) were loaded in the gel. Immunoblotting was done using anti-PrtV polyclonal antiserum. Lanes 1–3; whole cell lysates; lanes 4–6: culture supernatants from wild type *V*. *cholerae* El Tor O1 strains A1552, C6706, and P27459 respectively. (B) Ultrastructural analysis of *V*. *cholerae* by electron microscopy. An electron micrograph showing the flagella (open arrows) and OMVs (closed arrows). Bar, 500 nm. (C) PrtV association with OMVs from different *V*. *cholerae* isolates. Immunoblot analysis of OMVs from different *V*. *cholerae* isolates using PrtV polyclonal antiserum. Bacterial strains were grown at 30°C for 16 h and OMVs were isolated using the procedure described in Materials and Methods. 10 μl of OMV samples were loaded for immunoblot analyses and SDS-PAGE analyses by Coomassie blue staining. Lanes 1–6; OMVs from *V*. *cholerae* non-O1/non-O139 serogroup: V:52, V:5/04, V:6/04, KI17036, 93Ag19, and NAGV6; lanes 7–9: OMVs from *V*. *cholerae* O1 El Tor clinical isolates: P27459, C6706, and A1552; lane 10: OMVs from *V*. *cholerae* classical O1 strain 569B; lanes 11–13: OMVs from *V*. *cholerae* O1 environmental isolates: AJ4, AJ3, and AJ2. Lane 14, C6706 Δ*prtV* mutant.

In our earlier studies, we have shown that PrtV was secreted into culture supernatants of the *V*. *cholerae* wild type strain C6706 as a 102 kDa protein that due to autoproteolytic cleavages also resulted in two shorter forms (81 kDa and 37 kDa, respectively) with protease activity [9]. It was suggested that all three forms are physiologically important. Immunoblot analysis was used to confirm that PrtV is secreted by additional *V*. *cholerae* strains, i.e. the O1 El Tor strains A1552 and P27459 (Fig 1A). Electron microscopy analysis of strain C6706 revealed the presence of OMVs surrounding the bacterial cells (Fig 1B). As cell supernatants samples include not only soluble extracellular proteins, but also OMVs, we sought to assess if PrtV may be associated with OMVs in *V*. *cholerae*. Vesicles were isolated from overnight cultures (16 h) of a selection of strains as described in Material and Methods. The two forms of PrtV protein (81 kDa and 37 kDa) were detected by immunoblot in association with OMVs obtained from ten out of thirteen tested strains, i.e. from *V*. *cholerae* non-O1/non-O139 strainsV:5/04, V:6/04, KI17036, 93Ag19 and NAGV6 (Fig 1C, lanes 2–6); from O1 El Tor clinical isolates C6706 and A1552 (Fig 1C, lanes 8–9); from classical O1 strain 569B (Fig 1C, lane 10) and from O1 environmental isolates AJ4, AJ3 and AJ2 (Fig 1C, lanes 11–13). Interestingly, the non-O1/non-O139 *V*. *cholerae* strain V52 (Fig 1C, lane 1) and the O1 El Tor strain P27459 (Fig 1C, lane 7) have only one form of PrtV protein, the 81 kDa and 37 kDa form respectively. A Coomassie blue stained gel was shown to estimate the loading amount of each sample (Fig 1D) Based on these observations we propose that secretion of PrtV via OMVs may be common among *V*. *cholerae* strains.

### Detection of PrtV in association with purified OMVs of *V*. *cholerae* strain C6706

**Fig 2:**
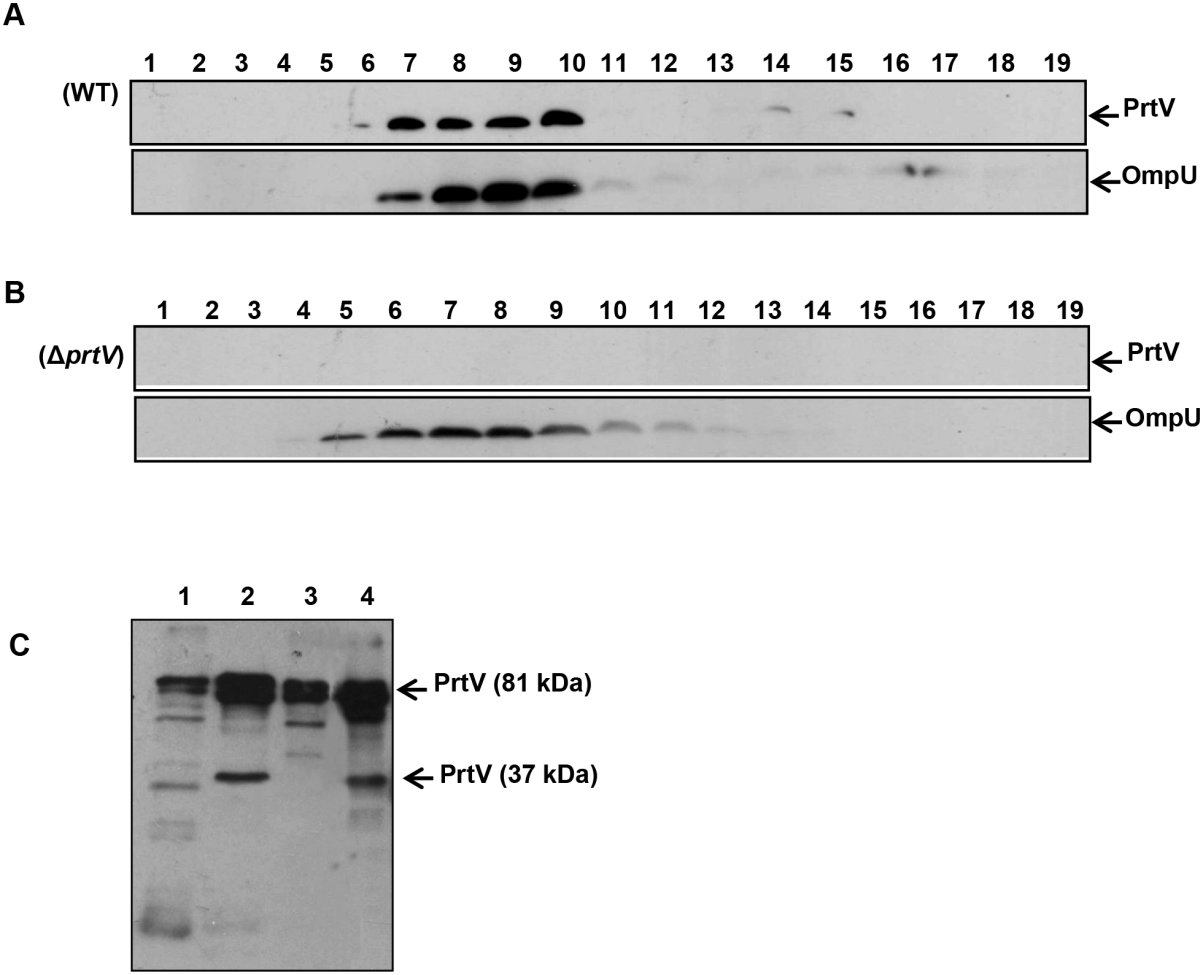
Presence of PrtV in OMVs from *V*. *cholerae* strain C6706. To detect the PrtV and the OmpU proteins in the density gradient fractions, immunoblot analyses were performed using polyclonal anti-PrtV and anti-OmpU antisera, respectively. (A) Immunoblot detection of PrtV (upper panel) and OmpU (lower panel) in density gradient fractions of OMVs from the wild type *V*. *cholerae* strain C6706. (B) Immunoblot detection of PrtV (upper panel) and OmpU (lower panel) in density gradient fractions of OMVs from the *prtV* mutant. (C) Immunobot detection of PrtV in the whole cell lysate (lane 1), culture supernatant before ultracentrifugation (lane 2), supernatant after the removal of OMVs (lane 3), and OMV sample (lane 4).

To confirm the secretion of PrtV in association with OMVs, vesicles from strain C6706 were isolated and purified using an Optiprep density gradient centrifugation, as described in Materials and Methods. Analysis of the Optiprep fractions by immunoblotting using anti-PrtV polyclonal antibody revealed the presence of PrtV protein in fractions 7–10 (Fig 2A, upper panel). The presence of outer membrane protein in these fractions was confirmed by immunoblotting using anti-OmpU polyclonal antibody (Fig 2A, lower panel). As a control experiment, we analysed OMVs from the *prtV* mutant derivative of C6706 using the same approach. The absence of PrtV in the density gradient fractions was confirmed by immunoblotting, whereas OmpU was detected in fractions 5–10 (Fig 2B, upper and lower panels, respectively). Based on these observations we concluded that PrtV was secreted in association with vesicles. To estimate what percentage of the secreted PrtV was with associated with OMVs (i) total secreted PrtV in the cell-free culture supernatants (before OMV isolation); (ii) soluble PrtV (supernatant after separation of the OMVs by ultracentrifugation); and (iii) OMV-associated PrtV (purified vesicle sample) were examined for three independent cultures of the strain C6706 by immunoblotting. The immunoblot analysis of a representative set of samples is shown in Fig 2C. For each culture the amount of total secreted PrtV was given arbitrarily the value of 100. The results, given as a percentage, indicated that most of the secreted PrtV was associated with vesicles (70 ± 5%), whereas a smaller fraction was present in a free soluble form in the supernatant (30 ± 5%).

**Fig 3:**
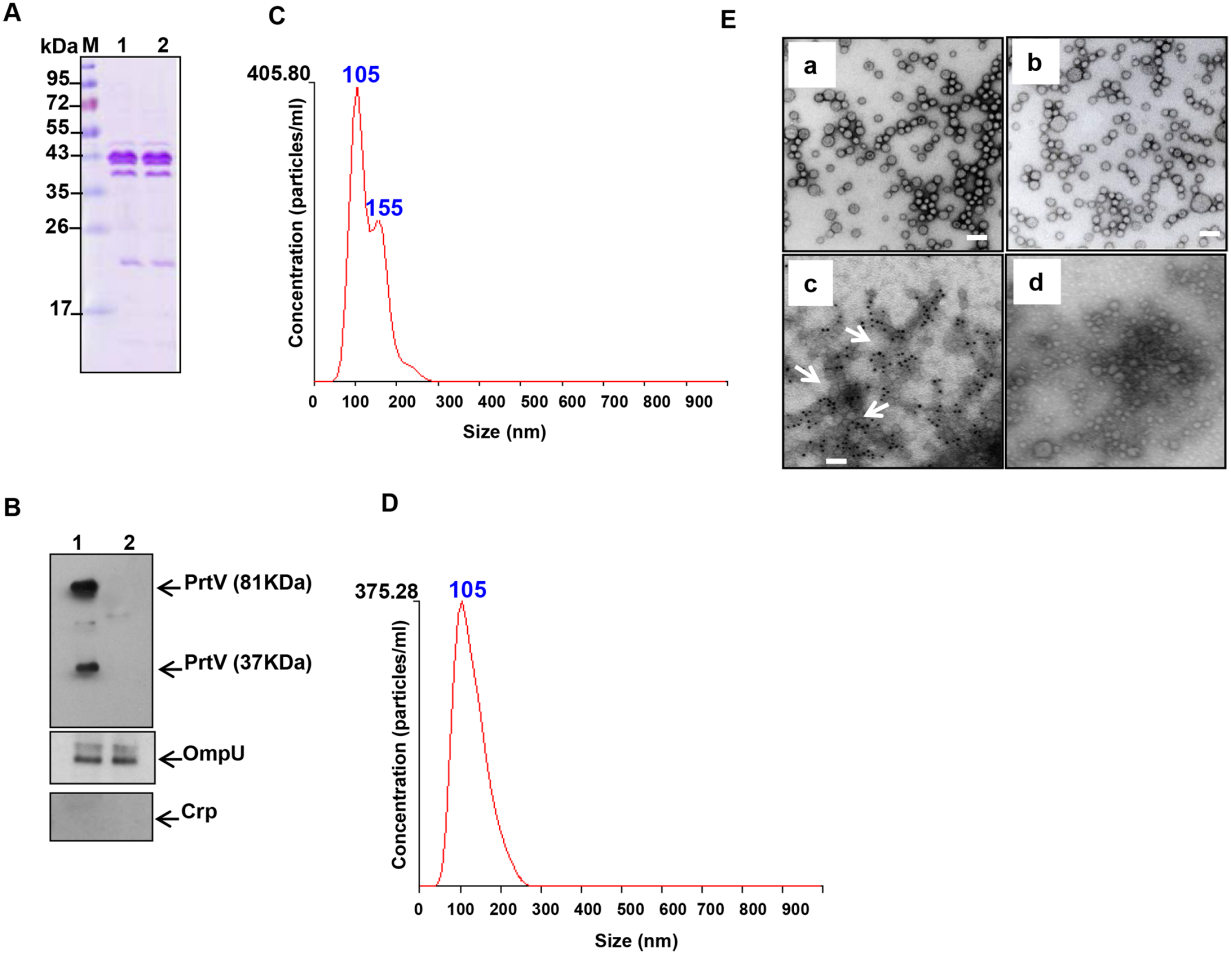
SDS-PAGE, immunoblot analyses, Nanoparticle tracking analysis and electron microscopic analyses of OMV samples from the wild type strain C6706 and the *prtV* mutant. (A) SDS-PAGE and Coomassie blue staining of OMVs samples from *V*. *cholerae* wild type C6706 (lane 1) and its derivative *prtV* mutant (lane 2). (B) Immunoblot analysis of PrtV protein in the OMV samples from the wild type strain C6706 (upper panel, lane 1) and the *prtV* mutant (upper panel, lane 2) using anti-PrtV polyclonal antiserum. Immunoblot analysis of OMV samples using anti-OmpU antiserum as a OMV marker (middle panel) and anti-Crp polyclonal antiserum as a cytoplasmic protein marker (lower panel). (C) Nanoparticle tracking analysis measurement of OMVs isolated from the wild type *V*. *cholerae* strain C6706 showing the sizes and total concentration of OMVs. (D) Nanoparticle tracking analysis measurement of OMVs isolated from the Δ*prtV* mutant showing the sizes and total concentration of OMVs. (E) Electron microscopy of OMVs from the wild type *V*. *cholerae* strain C6706 (a) and the *prtV* mutant (b). Immunogold labeling of OMVs from *V*. *cholerae* wild type strain C6706 (c) and the *prtV* mutant (d). White arrow points to gold particles associated with OMVs. Bars; 150 nm.

To further examine the OMVs from the wild type and *prtV* mutant strains, gradient fractions number 8 from C6706 and its *prtV* mutant were analysed. As shown by SDS-PAGE and Coomassie blue staining (Fig 3A), the OMV fraction from these two strains exhibited almost identical protein profiles. Immunoblotting confirmed the presence of PrtV in the C6706 OMV fraction only (Fig 3B, upper panel). As was observed in Fig 1C, we detected two PrtV bands at 81 kDa and 37 kDa, respectively. The 37 kDa might be an autoproteolytic form of PrtV protein in the OMVs. As judged by the protein profiles (Fig 3A) and the intensity of the OmpU band in the OMV samples from the wild type C6707 and the *prtV* mutant (Fig 3B, middle panel), the amount of OMVs released from the wild type and the *prtV* mutant was very similar. The total protein content of each OMV sample was measured using the Bicinchoninic Acid (BCA) assay kit as described in the materials and methods. It showed that OMVs from the wild type and Δ*prtV* mutant bacteria contain 1,090 μg/ml and 1,270 μg/ml protein, respectively. We used nanoparticle tracking analysis (NTA), a new method for direct, real-time visualization of nanoparticles in liquids [32]. In this system, OMVs can be observed by light scattering using a light microscope. A video was taken, and the NTA software can track the brownian movement of individual OMVs and calculate the size and concentration of OMVs. The amount of OMV particles measured by nanoparticle tracking analysis using the NanoSight equipment are shown in Fig 3C and 3D, the OMV samples from the wild type C6706 and Δ*prtV* mutant contained 7.5 x 10^12^/ml (Fig 3C) and 8.5 x 10^12^/ml OMV-particles (Fig 3D) respectively. The size distribution of OMVs isolated from both the wild type and Δ*prtV* mutant was in the 50–250 μm diameter range with the majority of the OMV particles at 105 μm from both the wild type and the Δ*prtV* mutant *V*. *cholerae* (Fig 3C and 3D). Interestingly, an extra peak representing 155 μm diameter sized OMVs was observed in the wild type OMV sample (Fig 3C). It could be considered that the soluble form of PrtV might form particles showing up as 155 nm on the nanoparticle tracking analysis since this method presumably cannot distinguish between different types of particles. The morphology of OMVs was examined by transmission electron microscopy, which also revealed similar sizes and morphology of OMVs from the wild type and the *prtV* mutant (Fig 3E, panels a and b). To test for possible contamination from lysed bacterial cells in these gradient fractions, immunoblotting was also carried out using antiserum against the cytoplasmic cAMP receptor protein (Crp). As this revealed no Crp reactive bands (Fig 3B, lower panel), we concluded that there was no detectable cytoplasmic contamination in these samples. In order to visualize the association of PrtV with OMVs, we carried out electron microscopy analysis and immunogold labeling using PrtV polyclonal antiserum. OMV-associated several gold particles were observed in the wild type strain, whereas no gold particles were associated with OMVs isolated from the *prtV* mutant (Fig 3E, panels c and d). Taken together, our results strongly support the idea that the PrtV protein is associated with OMVs released from *V*. *cholerae*.

### Full-length and auto-proteolytically digested forms of PrtV may be differentially associated with OMVs upon extracellular release

**Fig 4:**
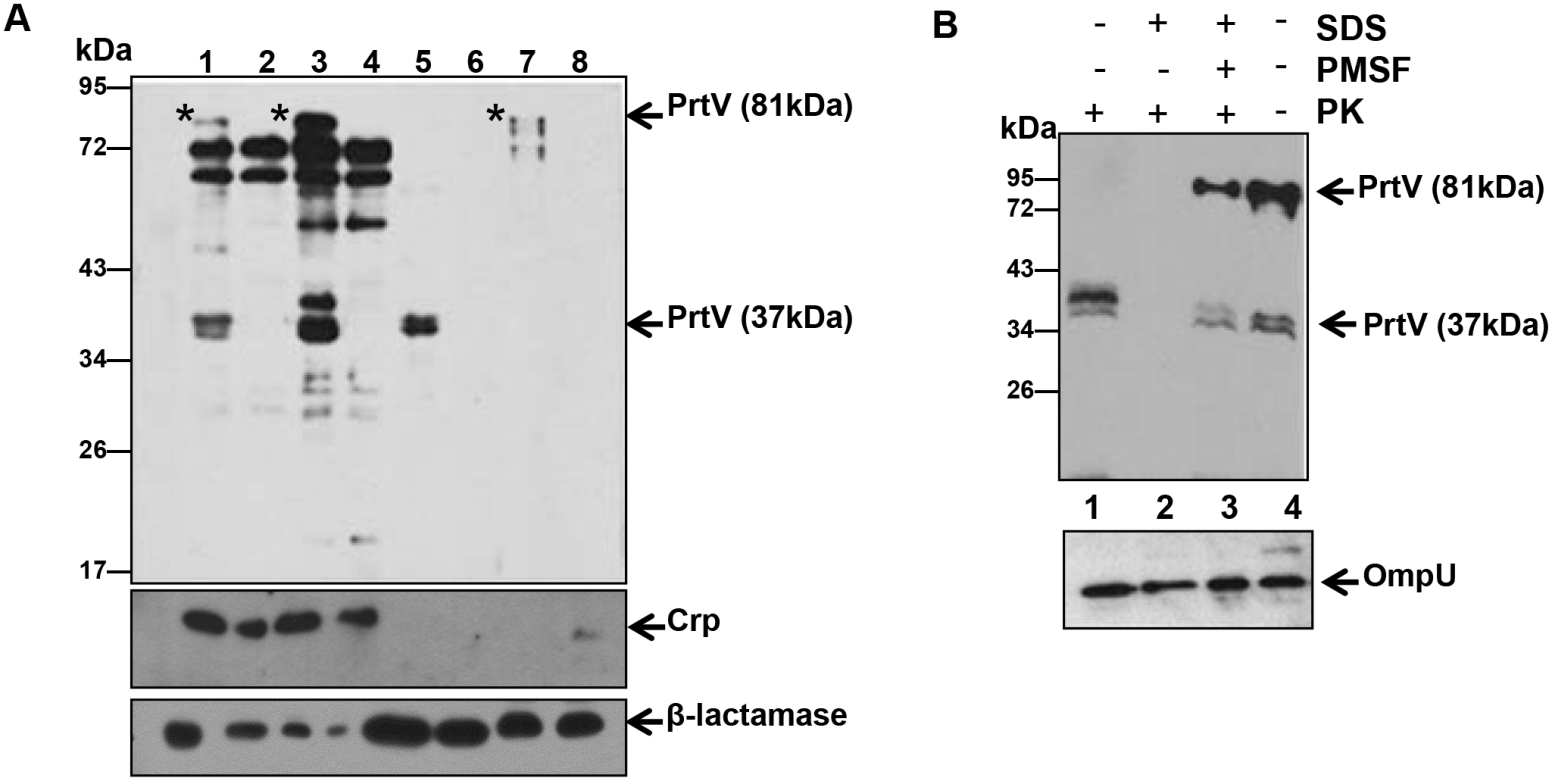
Immunoblot analysis of sub-cellular localization of PrtV protein in *V*. *cholerae*. (A) Immunoblot analyses of cell fractions from *V*. *cholerae* wild type strain C6706 (lanes 1, 3, 5, and 7) and the *prtV* mutant (lanes 2, 4, 6, and 8) using anti-PrtV serum (upper panel), anti-Crp antiserum (middle panel), and anti-β-lactamase antiserum (lower panel). Lanes 1 and 2: whole cell lysates; lanes 3 and 4, cytoplasmic fractions; lanes 5 and 6, periplasmic fractions; lanes 7 and 8, culture supernatants. Asterisks indicate the 81 kDa PrtV protein (B) Proteinase K susceptibility assay. OMVs from *V*. *cholerae* wild type strain C6706 were treated with 0.5 μg ml^-1^ of proteinase K (PK), 1% SDS and/or the proteinase K inhibitor PMSF (1 mM) as indicated. Samples were examined by immunoblot analysis using polyclonal anti-PrtV antiserum (upper panel). Lane 1: OMVs treated with only PK; lane 2: OMVs treated with SDS and PK; lane 3: OMVs treated with SDS, PMSF, and PK; lane 4: control OMVs. The same membrane was re-probed with OmpU antiserum as an internal control (lower panel).

To analyze how PrtV may be released from the bacterial cell via OMVs, we first determined the subcellular localization of the PrtV protein in the C6706/pBAD18 and C6706/pBAD::*prtV* strains using a fractionation assay. According to our findings, full-length, 81 kDa PrtV was present in the whole cell (Fig 4A, lane 1), cytoplasmic (Fig 4A, lane 3), and extracellular fractions (Fig 4A, lane 7), but not in the periplasmic fraction (Fig 4A, lane 5). Crp and β-lactamase were used as marker proteins to confirm the cytoplasmic and periplasmic content, respectively, of the fractions. The 37 kDa form of PrtV was abundant in the periplasmic fraction (Fig 4A, lane 5) and in the OMV fraction (Fig 3B, upper panel, lane 1), suggesting that the full-length protein is subject to proteolytic cleavage in the periplasmic space. Moreover, it could be hypothesized that the 37 kDa form is packaged as a part of the OMV luminal content during vesicle biogenesis. Our hypothesis was also supported by the Fig 2C results in which the 37kDa form is only in the OMV, not found in the supernatant.

To investigate how the different forms of PrtV may be carried by OMVs, a proteinase K protection assay was performed. When OMVs obtained from strain C6706 were incubated with proteinase K in the presence of SDS (1% w/v) to rupture the membrane of the vesicles, both forms of PrtV were proteolytically digested (Fig 4B, upper panel, lane 2). As an assay control, PMSF was used to inhibit proteinase K activity, resulting in slight proteolytic cleavage only of the two forms of PrtV (Fig 4B, upper panel, lane 3). Interestingly, only the full-length form of PrtV was digested by proteinase K in the absence of SDS, suggesting that the 37 kDa processed form was protected from proteinase K digestion by the vesicle structure (Fig 4B, upper panel, lane 1). As a control, OmpU immunoblot detection was shown (Fig 4B, lower panel). These results support our suggestion that the protected 37 kDa form may be carried inside the vesicle lumen upon release from the bacterial cells, whereas the full-length form might be associated on the surface of OMVs.

### PKD-domains in PrtV are required for its association with OMVs

The PrtV protein was shown to have two C-terminal two PKD-domains (PKD1 and PKD2) (http://merops.sanger.ac.uk/; http://pfam.sanger.ac.uk/ and [9]). NCBI conserved domains analysis (http://www.ncbi.nlm.nih.gov/Structure/cdd) suggested that the PKD-domains could function as ligand-binding sites for protein–protein or protein–carbohydrate interactions. PKD-domains are also found in some microbial collagenases and chitinases [33], as well as in archeael, bacterial and vertebrate proteins [34]. In our earlier studies, the results suggested that the PKD1 domain might have a role in Ca^++^ dependent stabilization of PrtV since secreted PrtV was not stable when the bacteria were grown in a defined medium with low concentration of Ca^++^ [13].

**Fig 5:**
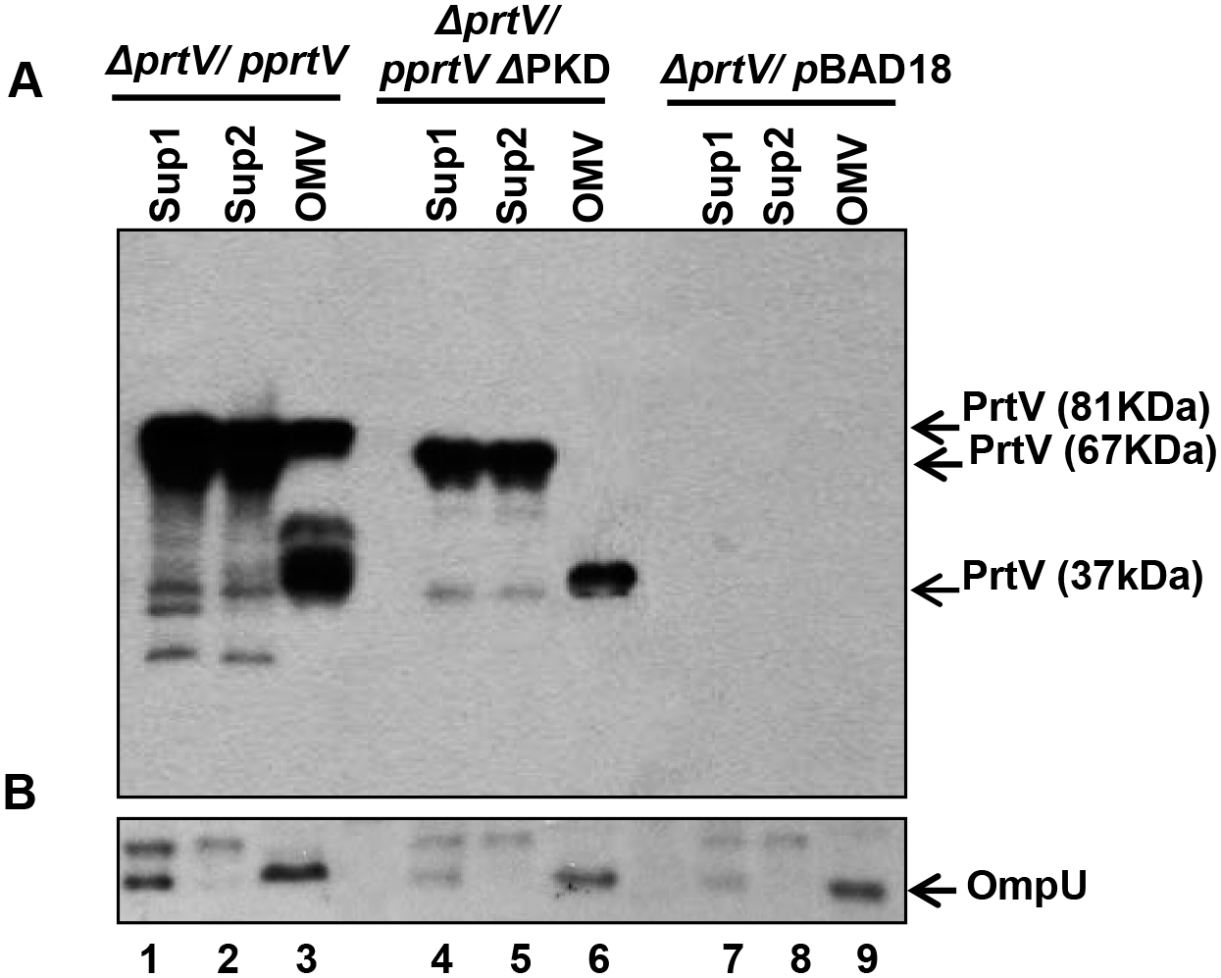
Immunoblot analyses of the wild type PrtV protein and its PKD domain deletion mutant in culture supernatants and OMV samples. Immunoblot analysis was performed using anti-PrtV antiserum (A) and anti-OmpU antiserum (B) with the following samples: lanes 1–3: *V*. *cholerae* Δ*prtV* strain carrying the cloned wild type allele of *prtV*; lanes 4–6: the Δ*prtV* strain carrying the cloned *prtV*ΔPKD allele; lanes 7–9: the Δ*prtV* strain carrying the pBAD18 cloning vector. Lanes 1, 4 and 7 were loaded with supernatant samples before ultracentrifugation (Sup1). Lanes 2, 5 and 8 were loaded with the supernatants after ultracentrifugation (Sup2). Lanes 3, 6 and 9 were loaded with the OMV samples.

In order to test the role of the PKD-domains in OMV-associated secretion of PrtV, we constructed expression plasmids encoding either the wild type *prtV* gene or a *prtV*ΔPKD1-2 allele. These clones and the empty pBAD18 vector were introduced into the *prtV* mutant of *V*. *cholerae* strain C6706. Bacterial culture supernatants before and after ultracentrifugation, and OMVs were isolated from these three strains, and the OMV-associated secretion of PrtV and PrtVΔPKD1-2 was analyzed by immunoblotting. Unlike full-length PrtV, the full-length PrtVΔPKD1-2 protein was not detected in association with OMVs (Fig 5A, lane 6) although the full-length PrtVΔPKD1-2 was observed in the supernatants, indicating that it was stable and translocated from the bacterial cells grown in LB media containing 50 μg/ml carbenicillin and 0.01% arabinose. Interestingly, the processed 37 kDa form of PrtV could be detected in OMVs regardless if the strain expressed wild-type PrtV or PrtVΔPKD1-2 (Fig 5A, lanes 3 and 6). As a control, OmpU immunoblot detection was shown (Fig 5B). Taken together, the findings suggested that the PKD domains might have a role for secreted full-length PrtV in its association with OMVs.

### Determination of mechanism of PrtV translocation

**Fig 6:**
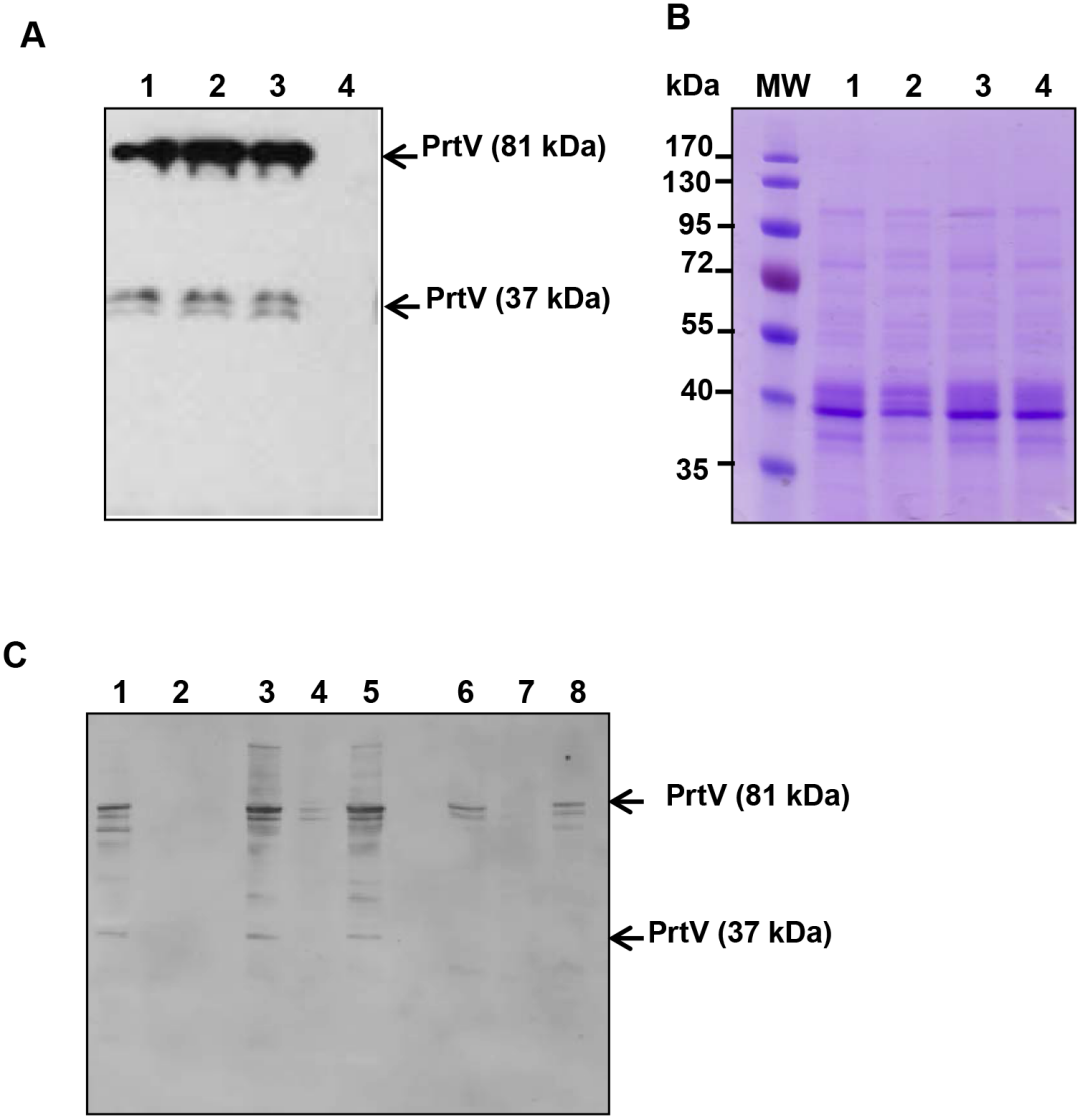
PrtV secretion in type I secretion mutants of *V*. *cholerae* O1 El Tor strain C6706. (A) PrtV secretion was analyzed using culture supernatants of *V*. *cholerae* wild type strain C6706 and its mutant derivatives. Lane 1: C6706; lane 2: Δ*tolC*; lane 3: Δ*hlyD*; lane 4: Δ*prtV*. (B) A Coomassie blue stained gel as a control for sample loading. Lane 1: C6706; lane 2: *ΔtolC*; lane 3: *ΔhlyD*; lane 4: *ΔprtV*. (C) PrtV secretion in type II secretion system mutants of *V*. *cholerae* O1 El Tor strains. Lanes 1 and 2: culture supernatants of wild type C6706 and Δ*prtV*; lanes 3, 4 and 5: culture supernatants of wild type 3083, Δ*epsC* and Δ*epsC* strain carrying the cloned *epsC* allele; lanes 6, 7 and 8: culture supernatants of wild type TRH7000, Δ*epsC* and Δ*epsC* strain carrying the cloned *epsC* allele.

Our findings prompted us to determine which secretion system might be involved in secretion of PrtV through the outer membrane and thereafter into the culture supernatant and/or OMVs. To test if the type I secretion system is needed for PrtV secretion, we constructed *tolC* and *hlyD* in-frame deletion mutants of *V*. *cholerae* wild type strain C6706 because the TolC and HlyD proteins are essential components of the type I secretion system of bacteria [35]. We compared secretion of PrtV in the *tolC* and the *hlyD* mutants with the wild type strain C6706 by immunoblot analysis. We observed no difference in the levels of secreted PrtV in the wild type and the mutants (Fig 6A, lanes 1–3). A Coomassie blue stained gel was included to verify equal sample loading (Fig 6B).

Similarly, to assess the possible involvement of the type II secretion system, we examined the secretion of PrtV in the *epsC* mutant in comparison with the O1 El Tor wild type *V*. *cholerae* strains 3083 and TRH7000. In previous studies, it was described that EpsC is required for the secretion of substrate proteins such as cholera toxin, protease(s), and chitinase(s) through the type II secretion system of *V*. *cholerae* [36]. As shown in Fig 6B, PrtV was not secreted from the *epsC* mutants of either the *V*. *cholerae* strain 3083 or TRH7000 (Fig 6B. lanes 4 and 7). Moreover, trans-complementation of *epsC* restored the secretion of PrtV into the culture supernatants (Fig 6B, lanes 5 and 8).

### OMV-associated PrtV is biologically active

**Fig 7:**
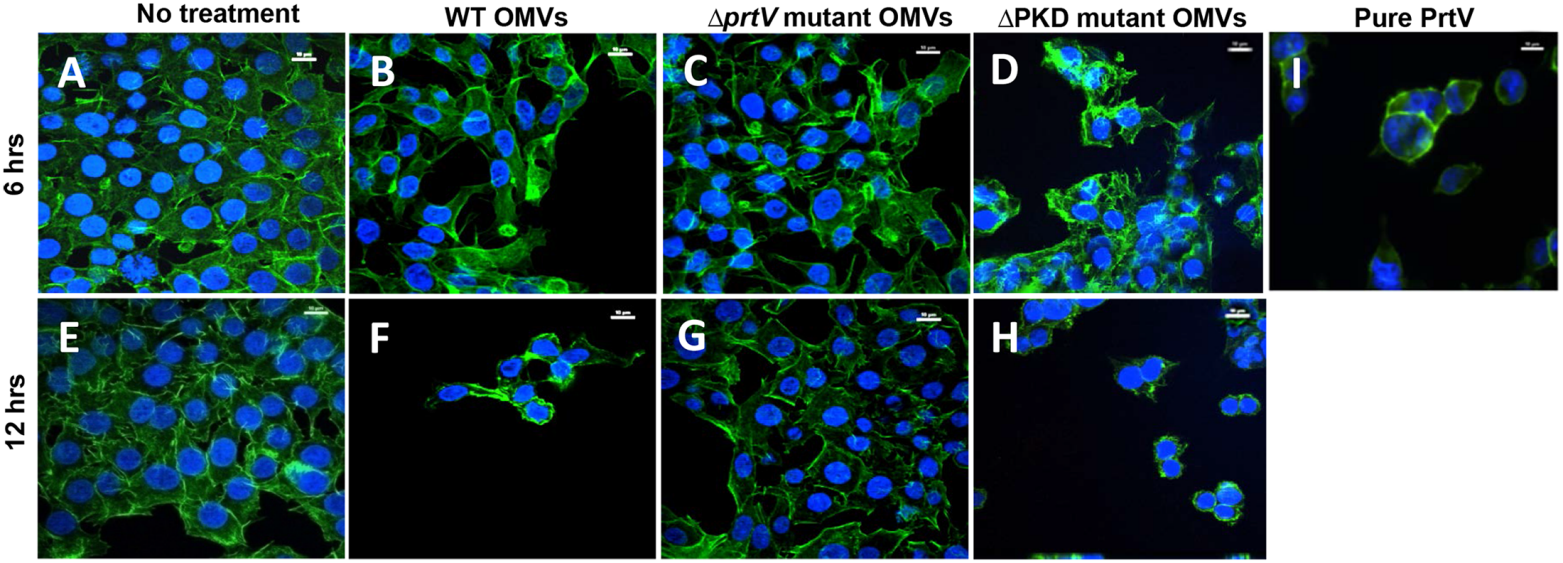
Analyses of biological activities of PrtV. HCT8 cells were treated with 50 μl OMVs (total protein concentration 60 μg/ml) from the wild type *V*. *cholerae* strain C6706 and the *prtV* mutant. Cells were treated with 20 mM Tris-HCl as a negative control (A, E), with OMVs from the wild type strain C6706 (B and F) or from the *prtV* mutant (C and G) or from the the *prtV* mutant/pΔPKD PrtV (D and H) or with 20 nM purified PrtV protein for 6 h as a positive control (I). The treatment was performed for 6 h (A, B, C, D) and for 12 h (E, F, G, H). Bars represent 10 μm.

In our earlier studies, we demonstrated that PrtV is an active protease as it is able to cleave proteins such as fibrinogen, fibronectin and plasminogen. We also showed that purified PrtV exhibited a dose-dependent cytotoxic activity towards mammalian cells [9]. To test the biological activity of vesicle-associated PrtV, we incubated HCT8 cells with OMVs obtained from the wild type strain C6706, the *prtV* mutant, and the strain expressed PrtVΔPKD. According to our findings, no apparent morphological changes of the epithelial cells were observed when the cells were treated with OMVs from these strains for 6 h (Fig 7B, 7C and 7D). However, after 12 h incubation, rounding and detachment of the HCT8 cells was observed when incubated with OMVs from both the wild type strain and the strain expressed PrtVΔPKD (Fig 7F and 7H). A similar result was obtained when the cells were treated with purified PrtV (20 nM) for 6 h (Fig 7I). In contrast, such morphological effects on the cells were not observed when the cells were treated with vesicles isolated from the Δ*prtV* mutant (Fig 7C and 7G) or with buffer (Fig 7A and 7E). Thus, based on these results we concluded that OMV-associated full length PrtV and PrtVΔPKD1-2 were both biologically active. Moreover, taking into consideration that OMVs isolated from the bacterial strain harboring only PrtVΔPKD alle contain only 37 kDa form suggesting that the biological activity that we observed might be mainly due to the action of 37 kDa form.

### *V*. *cholerae* OMVs are internalized into HCT8 cells independently of PrtV

**Fig 8:**
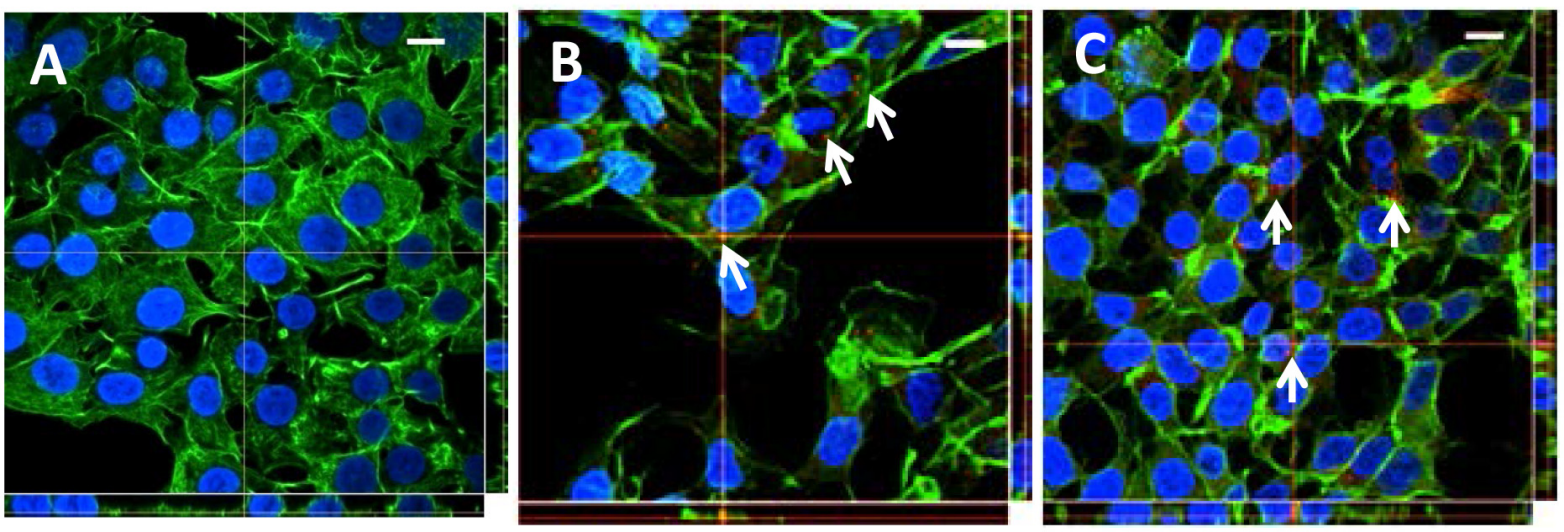
*V*. *cholerae* OMVs internalization into HCT8 cells. OMVs from the wild type strain C6706 and the *prtV* mutant were labeled with PKH26 red fluorescence marker and subsequently the HCT8 cells were treated for 6 hrs with buffer (A), PKH26-labeled OMVs from *V*. *cholerae* wild type strain C6706 (B) or with OMVs from the *prtV* mutant (C). After the treatment, cells were fixed and actin filaments and nuclei were stained with phalloidin and DAPI, respectively. Internalized OMVs are indicated with white arrows. Confocal Z-stack projections are shown in all images. The crosshairs indicate the positions of the xz and yz planes. Bars represent 10 μm.

To investigate if OMVs isolated from *V*. *cholerae* can internalize into HCT8 cells, confocal microscopy analyses were performed for the detection of the internalized vesicles. For this purpose, we labeled samples of OMVs isolated from the wild type *V*. *cholerae* strain C6706 and the *prtV* mutant containing 3.5 x 10^11^ and 4 x 10^11^ OMV-particles, respectively, with a red fluorochrome, PKH26. The use of this fluorescent marker to monitor cell trafficking and function has been well documented in earlier studies [37–40]. After incubating the cells with OMV samples (either WT C6706 or the *prtV* mutant) for 1 h, we observed that several wild type OMVs, appearing as red dots surrounding the nuclei of the effected cells (Fig 8B). Similarly, OMVs obtained from the *prtV* mutant were internalized into the HCT8 cells (Fig 8C). Based on these observations we concluded that there was indeed internalization *V*. *cholerae* OMVs were internalized into the HCT8 cells regardless of the presence of PrtV.

### OMV-associated PrtV protease contributes to *V*. *cholerae* resistance to the host antimicrobial peptide LL-37

**Fig 9:**
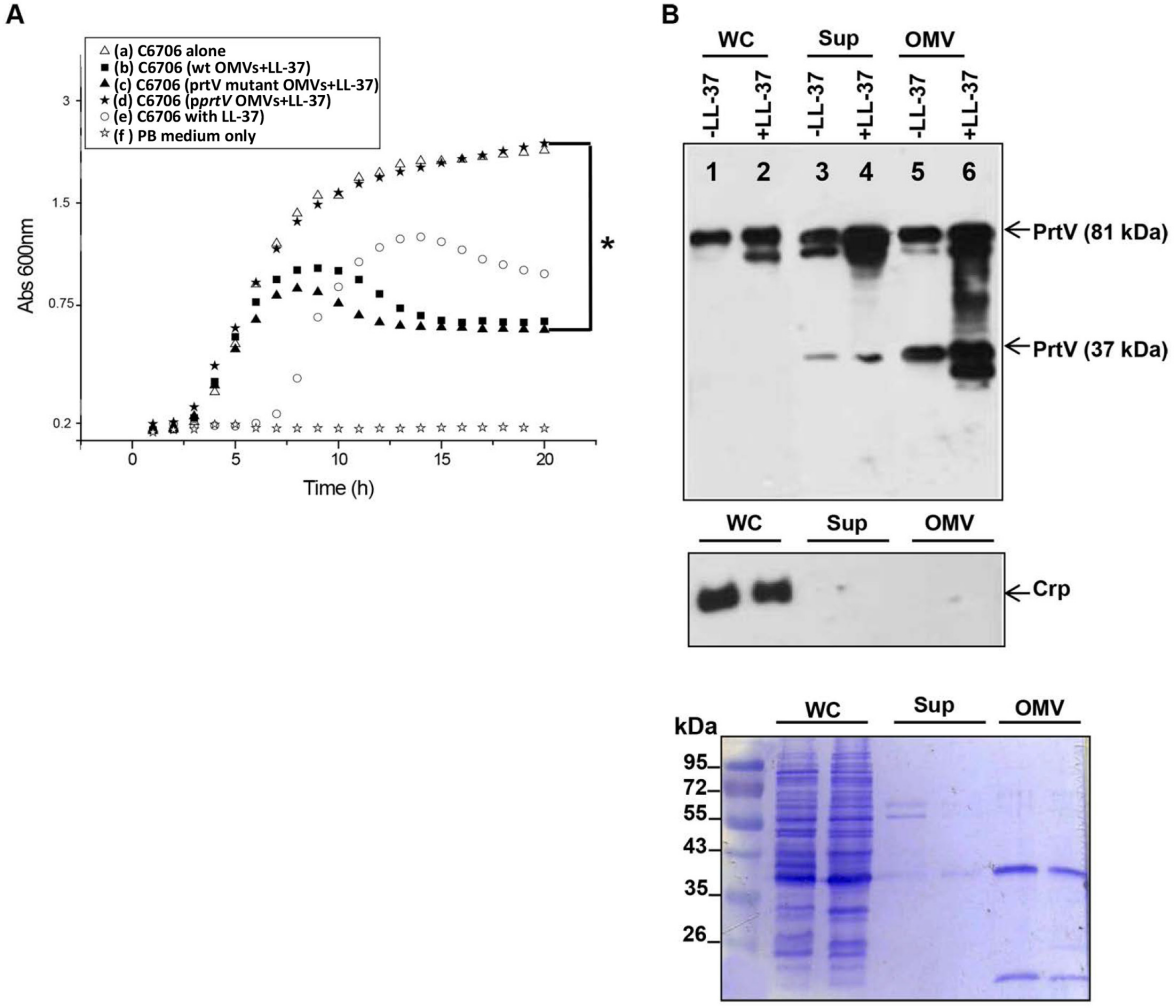
Analysis of role of OMV-associated PrtV in LL-37 resistance. (A) PrtV protein contributes to bacterial resistance against LL-37. *V*. *cholerae* O1 El Tor strain C6706 was grown in the presence of OMVs pre-incubated with a sub-lethal concentration (25 μg/ml) of the antimicrobial peptide LL-37. Bacterial growth was monitored spectrophotometrically at 600 nm for 20 h at 30°C. C6706 was grown under the following conditions: Open triangle (a) C6706 without LL-37, closed rectangle (b) in the presence of wild type OMVs and LL-37, closed triangle (c) in the presence of Δ*prtV* OMVs and LL-37, closed star (d) in the presence of OMVs from Δ*prtV* strain carrying the cloned wild type allele of *prtV* in pBAD18 and LL-37, open circle (e) in the presence of LL-37 and no OMVs, open star (f) no bacteria, poor broth (PB) medium only. A statistically significant growth difference (* = P<0.05) was observed between curves (d) and (c). (B) The antimicrobial peptide LL-37 induces more PrtV secretion in *V*. *cholerae*. Immunoblot analysis of expression and secretion of PrtV in response to the antimicrobial peptide LL-37. *V*. *cholerae* O1 El Tor strain C6706 was grown at 30°C in the presence and absence of a sub-lethal concentration of LL-37 (25 μg/ml) and samples were collected at OD_600_ 2.0. Immunoblot was performed using anti-PrtV polyclonal antiserum (upper panel) and anti-Crp polyclonal antiserum to detect Crp, a cytoplasmic protein marker (middle panel). Lanes 1 (without LL-37) and 2 (with LL-37): whole cell lysates; lanes 3 (without LL-37) and 4 (with LL-37): culture supernatants; lanes 5 (without LL-37) and 6 (with LL-37) OMVs. Lower panel: SDS-PAGE and Coomassie blue staining of samples from (A) and (B).

AMPs are believed to be a first line defense molecules against different pathogenic microorganisms, including bacteria, viruses and fungi [41]. However, different pathogenic bacteria may also be able to sense and resist AMP-mediated killing during the course of infection [42]. To investigate the role of OMV-associated PrtV in bacterial protection against AMPs, OMVs from the wild-type *V*. *cholerae* strain C6706 or its *prtV* mutant derivative were co-incubated for 1 h with a sub-lethal concentration (25 μg/ml) of LL-37, a human amphipathic peptide. The wild type *V*. *cholerae* strain C6706 was grown in LB medium with LL-37, without LL-37, and with OMVs pre-incubated with LL-37. The growth of bacteria was monitored for 20 h at 30°C (Fig 9A). The growth of the wild type C6706 strain was reduced in the presence of LL-37 in comparison to without LL-37 and characterized by a long lag-phase of growth (Fig 9A, compare a and e). Interestingly, when the bacteria were grown in the presence of OMVs isolated from the PrtV over-expressing strain, the growth was not affected by LL-37 (Fig 9A, compare a and d). It suggested that OMV-associated PrtV might degrade LL-37 and protect the bacteria against LL-37. The long lag-phase growth pattern in the presence of LL-37 was not observed when the bacteria were grown either in the presence of OMVs isolated from the wild type *V*. *cholerae* strain C6706 or OMVs from the Δ*prtV* mutant (Fig 9A, compare e with b and c). It indicated that the restoration of a shorter lag-phase by addition of OMVs was not PrtV dependent. We also tested the effect of LL-37 on the expression of PrtV in the wild type C6706 strain by immunoblot analysis. There was no obvious difference of PrtV levels in the whole cell lysates from the C6706 strain with or without LL-37 treatment (Fig 9B, upper panel, lanes 1 and 2). However, it may be that the LL-37 treatment permeabilizes cells, releasing the PrtV into the supernatant. There was enhanced detection of PrtV in supernatants (Fig 9B, upper panel, compare lanes 3 and 4) and associated with OMVs (Fig 9B; upper panel, compare lanes 5 and 6) when the cells were grown in the presence LL-37, suggesting that *V*. *cholerae* releases increased amounts of free and OMV-associated PrtV protein in response to the antimicrobial peptide LL-37. Hence, based on these findings we concluded that OMV-associated PrtV may contribute to *V*. *cholerae* resistance to the human antimicrobial peptide LL-37.

## Discussion

In our earlier studies, we discovered that the PrtV protease is essential for *V*. *cholerae* environmental survival and protection from natural predator grazing [8]. Further studies showed that PrtV has proteolytic activity and can effectively degrade human blood plasma components and induce a dose-dependent cytotoxic effect in the HCT8 cell line [9]. Although the biological role of PrtV protein as a secreted protease was characterized, the mechanism or pathway by which PrtV protein might be released into the culture supernatant was not yet clarified. In this study, we showed that the PrtV protein is efficiently secreted into the culture supernatants by the type II secretion system in multiple *V*. *cholerae* strains. Additionally, PrtV association with OMVs was observed in samples from several different serogroups of *V*. *cholerae*, suggesting that OMV-associated secretion of this protein is commonly occurring in *V*. *cholerae*.

Full-length PrtV protein contains 918 amino acids and consists of one M6 peptidase domain, a zinc- binding domain, and two C-terminal Polycystic Kidney Disease domains (PKD1 and PKD2) [9]. While the PKD-domain has been also found in bacterial collagenases [43], proteases [44], and chitinases [45]. The functional significance of the PKD1 and PKD2 domains in *V*. *cholerae* PrtV is not yet understood. Our recent studies suggested that the PKD1 domain in *V*. *cholera*e PrtV (residues 755–838) might have a role in stabilization of PrtV protein when the bacterial strains were grown in Ca^++^ depleted media. In this study, we observed that the PKD-domains of PrtV were essential for the association of full-length PrtV with OMVs. Outer membrane proteins or LPS on the surface of OMVs might be the factors, which bind to PKD-domain(s) of the PrtV protein, as it was suggested that the PKD-domain has a role in protein-protein interactions or protein-carbohydrate interactions. Currently, we are analyzing how the PKD-domains of the PrtV protein interact with the surface of the OMVs.

In our recent study, the crystal structure of the PKD1 domain from *V*. *cholera*e PrtV (residues 755–838) revealed a Ca(^2+^)-binding site which controls domain linker flexibility, presumably playing an important structural role by providing stability to the PrtV protein [13].

In the type II secretion system (T2SS), proteins are first translocated via the inner membrane to the periplasmic space and are then transported across the outer membrane by a terminal branch of the T2SS [46,47]. In this study, we showed that PrtV is translocated to the extracellular milieu via the type II secretion system. In general, type II secretion substrates are not easily detectable during transit in the periplasm. In the case of PrtV, we could not detect the full-length form of the protein in the periplsm, but a relatively high amount of a periplasmic truncated species was observed. Therefore, proteolytic processing might occur in the periplasmic space resulting in a truncated PrtV protein that is packaged into the OMVs and released from the bacterial cell by entrapment inside the lumen of the OMVs. It might be that there is a tight binding/attachment between the processed 37 KDa form of PrtV and an inner-leaflet component of the outer membrane. Further studies are needed to assess this possibility.

OMVs have been proposed to be vehicles for virulence factor delivery to the host cells and play an active role in bacterial pathogenesis [15–19,48]. In order to monitor the activity of OMV-associated PrtV, we treated a human colon carcinoma cell line (HCT8) with OMVs from the wild type strain C6706 and from the *prtV* mutant. The HCT8 cells treated with OMVs from wild type strain showed significant morphological changes, whereas cells treated with OMVs from the *prtV* mutant remained unaltered. Taken together, the results suggest that a truncated 37 kDa PrtV protein was enough to cause biological effects on the target host cells since it was the main form of OMVs associated PrtV. In our earlier studies, we demonstrated that the 102 kDa PrtV protease is secreted from *V*. *cholerae* cells and undergoes several steps of auto-proteolytic cleavage resulting in an enzymatically active 37 kDa form (amino acids 106–434) and a 18 kDa form (amino acids 587–749) [9]. The 37 kDa form was shown to contain the predicted catalytic domain with Zn^2+^ binding site and it displayed proteolytic activity when it was measured using fluorescein-labeled gelatin. In an assay using human blood plasma as a source of potential substrates for the PrtV protease, we observed that the extracellular matrix proteins fibronectin, fibrinogen, and plasminogen were degraded by the PrtV protein [9]. The mechanism(s) of interaction between PrtV and these substrates in vivo remain unknown. OMVs might be a delivery system of biologically active PrtV to interact with extracellular matrix proteins and to interfere with innate immune responses in wound or intestinal infections. In addition, the 37 kDa form might be protected by OMV structural components against other proteases secreted by *V*. *cholerae* in the culture supernatant since free soluble 37 kDa form was rarely detectable in the OMV free culture supernatants. Furthermore, our data suggest that OMV-associated PrtV protease may contribute to *V*. *cholerae* resistance against host AMP. Presumably the OMV-associated PrtV has the potential to degrade the LL-37 antimicrobial peptide and thereby enhance bacterial survival by avoiding this innate immune defense of the host.

## Materials and Methods

### Bacterial strains and plasmids

The *Vibrio cholerae* strains used in this study are listed in Table 1. The bacterial strains were grown in Luria-Bertani (LB) liquid medium at 37°C or at 30°C for 16 hours. Antibiotics were used at the following concentrations when required: carbenicillin (Cb), 50 μg/ml rifampicin (Rif), 100 μg/ml streptomycin (Sm). The mutants *(ΔprtV*, *ΔtolC* and *ΔhlyD*) were constructed by deleting the entire open reading frame using previously described methods [8,49]. Primers used in this study are listed in Table 2.

**Table 1:** Bacterial strains used in this study.

Strains	Relevant Genotype/Phenotype	Reference/Source
*E*. *coli* DH5α	F^−^,ø80d*lacZ*ΔM15,Δ(*lacZYA-argF*) U169 *deoR*, *recA*1, *endA*1, *hsdR*17 (rk^-^,mk^+^), *phoA*, *supE*44, ʎ^-^, *thi*-1, *gyrA*96, *relA*1	[57]
*E*. *coli* SM10ʎ*pir*	*thi thr leu tonA lacY supE recA*::RP4-2 Tc::Mu Km ʎ*pir*	[58]
*V*. *cholerae* A1552	O1 El Tor, Inaba, Rif^R^	[48]
*V*. *cholerae* C6706	O1 El Tor, Inaba, Sm^R^	[8]
*V*. *cholerae* P27459	O1 El Tor, Inaba, Sm^R^	[59]
*V*. *cholerae* 569B	O1 Classical, Inaba	[60]
*V*. *cholerae* C6706 *ΔprtV*	*ΔprtV* derivative of C6706	[8]
*V*. *cholerae* C6706 *ΔtolC*	*ΔtolC* derivative of C6706	This study
*V*. *cholerae* C6706 Δ*hlyD*	*ΔhlyD* derivative of C6706	This study
*V*. *cholerae* C6706 Δ*pkd*	*Δpkd* derivative of C6706	This study
*V*. *cholerae* 3083	O1 El Tor, Ogawa	[61]
*V*. *cholerae* 3083 *ΔepsC*	*ΔepsC* derivative of 3083	[36]
*V*. *cholerae* V52	O37 serotype	[62]
*V*. *cholerae* V:5/04	non-O1 non-O139	[63]
*V*. *cholerae* V:6/04	O9 serotype	[62]
*V*. *cholerae* 93Ag19	O14	Argentina, 1993
*V*. *cholerae* NAGV6	non-O1 non-O139	Thailand, 1995
*V*. *cholerae* KI17036	non-O1 non-O139	Sweden, 2006
*V*. *cholerae* AJ-2	O1, Inaba	Japan, 1981
*V*. *cholerae* AJ-3	O1, Inaba	Japan, 1981
*V*. *cholerae* AJ-4	O1, Ogawa	Japan, 1981

**Table 2:** Plasmids and primers used in this study.

**Plasmids**	**Relevant Genotype/Phenotype**	**Reference/Source**
pGEM-Teasy	Cb^R^ TA-cloning vector plasmid	Promega
pCVD442	Cb^R^ positive selection suicidal vector plasmid	[64]
p*ΔtolC*	pCVD442-based suicide plasmid for generating Δ*tolC*, Cb^R^	This study
p*ΔhlyD*	pCVD442-based suicide plasmid for generating *ΔhlyD*, Cb^R^	This study
p*Δpkd*	pCVD442-based suicide plasmid for generating *Δpkd*, Cb^R^	This study
pMMB66EH	*epsC* complementation plasmid, Cb^R^	[65]
**Primer**	**Sequence**	**Source**
TolC-A	*5′CGCTCTAGAGGATCTGTTCGATGATCAC3′*	This study
TolC-B	*5′TGTTAGTTTATAGTGGATGGGCATCGGTCCTATTCCTGAC3′*	This study
TolC-C	*5′CCCATCCACTATAAACTAACAGTCGCGAAGAAGTAATCCATCTC3′*	This study
TolC-D	*5′CGCTCTAGACTTAACCATGCAGCAGAG3′*	This study
HlyD-A	*5′CGCTCTAGAGGGAAATGGAGGCTAATTTTG3′*	This study
HlyD-B	*5′CCCATCCACTATAAACTAACACTGCGGCGAAAGGCCTATTCA3′*	This study
HlyD-C	*5′TGTTAGTTTATAGTGGATGGGGCCCTATGAAACGTTGGATTG3′*	This study
HlyD-D	*5′CGCTCTAGACACTCGGAGTGGAGTAATACG3′*	This study
*prtV*-F	*5′CGCTCTAGACACTCGGAGTGGAGTAATACG3*	[8]
*prtV*-R	*5′AATAAAGCTTTTCGCATTGGCATGAGCCTTA3′*	[8]
*prtV*ΔPKD-F	*5′CGCGCGTCTAGACACCTTAAATAAGGAAATATT3*	This study
*prtV*ΔPKD-R	*5′CGCGCGAAGCTTTTAATTTTCCGTGGTGACTTT3′*	This study

### Isolation of OMVs from *V*. *cholerae* strains

OMVs were isolated from bacterial culture supernatants as described previously [16]. Briefly, bacterial cultures grown at 30°C for 16 h were centrifuged at 5000 x *g* for 30 min at 4°C. Then the supernatants were filtered through a 0.2-μm pore size sterile Minisart High Flow syringe filter (Sartorius Stedim) and ultracentrifuged at 100,000 x *g* for 2 h at 4°C in a 45 Ti rotor (Beckman). The vesicle pellet was resuspended in 20 mM Tris-HCl pH 8.0 buffer and the suspension was used as the crude OMV preparation. Samples were analysed by SDS-PAGE, electron microscopy and immunoblotting.

### Measurement of protein concentration and number of vesicle particles in OMV preparations

Bicinchoninic Acid (BCA) Assay kit (Thermo Scientific Pierce, Rockford, IL) was used to measure total protein content. A NanoSight NS500 instrument (Malvern Ltd, Worchestershire, UK) was used for determination of OMV particle size and concentration as described [50]. Briefly, samples were diluted 1:2,500 in Tris-HCl pH 8.0 buffer and loaded in the sample chamber. Videos were recorded for 60s and size of individual OMVs and total amount of OMV particles were analyzed by Nanoparticle Tracking Analysis software (NanoSight Ltd.). All measurements were performed at room temperature.

### OMVs purification by density gradient centrifugation

Optiprep density gradient purification was done as described previously [24]. Crude OMVs samples suspended in 20 mM Tris-HCl pH 8.0 were added on the top of gradient layers and centrifuged at 100,000 x *g* for 180 min at 4°C. After ultracentrifugation, the fractions were sequentially collected from the top of the tube and were analyzed by SDS-PAGE and immunoblotting.

### SDS-PAGE and immunoblot analysis

Bacterial strains were grown at 30°C in LB medium to an OD_600_ of 2.0 or for 16 h. Bacteria were harvested by centrifugation at 18000 x *g* for 5 minutes. The resulting pellet was suspended in 20 mM Tris-HCl (pH 8.0) buffer containing 8% SDS and 5% 2-mercaptoethanol. The supernatant sample was precipitated 1:4 with 50% (w/v) trichloroacetic acid (TCA) and incubated on ice for 15 minutes and subsequently centrifuged at 18,000 x *g* for 15 minutes at 4°C. The pellet was washed twice with ice-cold acetone, then air dried for 10 minutes at room temperature and resuspended in 20 mM Tris-HCl (pH 8.0) buffer containing 8% SDS and 5% 2-mercaptoethanol. The protein samples were separated by sodium dodecyl sulfate-13% polyacrylamide gel electrophoresis (SDS-PAGE) and blotted onto a PVDF membrane [51]. Immunoblot analysis was performed as described [52]. Full-length 81 kDa PrtV or cleaved 37 kDa were identified using polyclonal rabbit anti-PrtV antiserum with the final dilution of 1:20,000 [10]. The anti-OmpU (1:10,000 dilution) [53] antiserum was used to detect OmpU, a marker for the OMVs, anti-β-lactamase antiserum (1:3,000) was used as a periplasmic protein marker after the cell fractionation and anti-Crp (1:5,000) antiserum [54] was used to detect Crp, a cytoplasmic protein marker. ECL anti-rabbit IgG, horseradish peroxidase-linked whole antibody (GE Healthcare) was used as a secondary antibody at a final dilution of 1:20,000. The immunoblot detection was performed using the ECL+ chemiluminescence system (GE Healthcare, United Kingdom) and the level of chemiluminescence was measured by using a luminescent image analyzer LAS4000 IR multi colour (Fujifilm).

#### Construction of expression plasmids

DNA fragments containing the wild type *prtV* gene and the *prtV*ΔPKD gene were amplified by PCR using C6706 chromosomal DNA as a template and primers listed in Table 2. The PCR products were purified from the gel and ligated into the pGEM-T Easy vector (Promega). After transformation into the *Escherichia coli* strain DH5α, plasmids were isolated with a Qiaprep Spin Miniprep kit (Qiagen). The *prtV* and the *prtV*ΔPKD gene fragments were digested with *Xba*I and *Hind*III enzymes and cloned into the pBAD18 arabinose-inducible vector plasmid. The pBAD18-*prtV* and the pBAD18-*prtV*ΔPKD vectors were then electroporated into the Δ*prtV* mutant of *V*. *cholerae* strain C6706. As a negative control, the expression vector pBAD18 without an insert was introduced into the mutant strain.

### Electron microscopy and immunogold labeling

For the electronmicroscopic analysis, the OMV samples were stained with 0.1% uranyl acetate, then placed on carbon-coated Formvar grids, and examined under an electron microscope. Electron micrographs were taken with a JEOL 2000EX electron microscope (JEOL Co., Ltd., Akishima, Japan) operated at a voltage of 100 kV.

Immunogold labeling of OMV samples was performed as described previously [26]. Briefly, 50-μl (~3 μg of protein) of the OMV sample was treated with anti-PrtV polyclonal anti-serum diluted in phosphate-buffered saline (PBS) for 30 min at 37°C. The OMVs were then separated from the serum by centrifugation at 100,000 × *g* for 2 h at 4°C. The OMV samples were washed three times with PBS. After washing, the OMV samples were incubated with the colloidal gold probe suspension (Wako Pure Chemical Industries Ltd., Osaka, Japan) and the sample was kept at room temperature for 30 min. The unbound gold particles were removed by subsequent washing with PBS. After washing, the OMV samples were stained with 0.1% uranyl acetate on carbon-coated Formvar grids and electron microscopic analysis was performed.

### Proteinase K susceptibility assay

The assay was carried out as described previously [21,47]. Briefly, OMVs (150 μg/ml total protein concentration) were treated with proteinase K (0.5 μg ml^−1^) either in the absence or presence of 1% SDS and incubated at 37°C for 30 min in 20 mM Tris HCl (pH 8.0). To neutralize the activity of proteinase K, 1 mM phenylmethylsulfonyl fluoride (PMSF) was added and the sample was incubated on ice for 30 min. The samples were analyzed by SDS-PAGE and immunoblot analyses using anti-PrtV and anti-OmpU antiserum.

### Sub-cellular fractionation

*V*. *cholerae* O1 El Tor strain C6706 was grown at 37°C in LB medium to OD _600_ 2.0. Cell fractionation to obtain sub-cellular fractions was performed as described previously [26,55].

Samples were analysed by immunoblot with anti-PrtV, anti-β-lactamase and anti-Crp polyclonal rabbit antibodies.

### Labeling of OMVs using the red fluorescent dye PKH26

OMVs isolated as described above from the wild type strain C6706 and Δ*prtV* mutant were labeled using the red fluorescent dye PKH26 (Sigma). To maximize the dye solubility and staining efficiency, the vesicle pellet was suspended in 500 μl of Diluent C, provided by the manufacturer. PKH26 solution (2x10^-6^ M of PKH26 in 500 μl of Diluent C) was added to 500 μl of OMVs and mixed; the excess of unbound PKH26 dye was removed by two-step centrifugations (100,000 x *g*, 30 minutes). After washing, the labeled vesicles were re-suspended in 20mM Tris HCl pH 8.0.

### Cell line, culture conditions, and media used for the growth of cells

Human ileocecal colorectal adenocarcinoma (HCT8) cells (ATCC number CCL-244) were cultured in RPMI 1640 medium (Gibco) supplemented with 10% heat-inactivated fetal calf serum, 1 mM sodium pyruvate, 2 mM L-glutamine, 10 mM HEPES buffer solution, 100 μg/ml streptomycin and 100 U/ml penicillin. The cells were cultivated at 37°C in 5% CO_2_ atmosphere.

### OMV-associated PrtV activity assay

24-well plates (Thermo Scientific Nunclon) were seeded with HCT8 cells and the cells were grown to 50% confluence. The seeded HCT8 cells were incubated with 50 μl of OMVs or Tris-HCl buffer (as a control) for 12 h. 2% paraformaldehyde in PBS (pH 7.3) was used to fix the cells for 10 min. After fixation, the cells were washed with PBS and incubated with 0.1 M glycine at room temperature for 5 min. Subsequently, the cells were washed with PBS and permeabilized with 0.5% Triton X-100 (Sigma-Aldrich). Actin filaments and nuclei were stained using Alexa Fluor 488 phalloidin (Molecular Probes) containing 1% BSA (Sigma-Aldrich) and DAPI (Sigma-Aldrich) respectively. Cells were mounted in a fluorescence mounting medium (DAKO), analyzed with a NIKON Eclipse 90i microscope, and photographed using a Himamatsu BW digital camera (12 bit) (Hamamatsu, Hamamatsu City, Japan).

### Confocal microscopy

Cells were mounted with a fluorescence mounting medium (DAKO) containing antifade. Confocal microscopy was performed using a Nikon D-Eclipse C1 Confocal Laser with a NIKON Eclipse 90i Microscope. Images were taken using a NIKON color camera (24 bit) with Plan Apo NIKON 60X objective. Fluorescence was measured at 488 nm (FITC-CtxB and Alexa Fluor 488-phalloidin, green), 405 nm (DAPI, blue) and 543 nm (rhodamine isothiocyanate B-R18, red). Z-stack images were captured by using EZ-C1 3.80 imaging software.

### Antimicrobial peptide susceptibility assay

OMVs from *V*. *cholerae* O1 El Tor C6706 and its *prtV* mutant derivatives were isolated as described above. Isolated OMVs were diluted using 20 mM Tris HCl (pH 8.0) buffer to obtain physiological concentration (1x) [adjusted to the initial bacterial culture volume]. The OMV samples were co-incubated with 25 μg/ml of LL-37, a sub-lethal concentration, for 1 h at 37°C prior to liquid growth inhibition assay using the *V*. *cholerae* O1 El Tor C6706 strain. Liquid growth inhibition assays [56] were performed in PB medium (Poor Broth Medium: 0.5 M NaCl, 1% bactotryptone, pH 7.5) and bacterial growth was monitored spectrophotometrically at 600 nm for 20 h at 30°C using a TECAN multiscan microplate reader. To determine the effect of antimicrobial peptide LL-37 on PrtV expression and secretion, *V*. *cholerae* O1 El Tor C6706 were grown at 30°C in the presence of sub-lethal concentration of LL-37 (25 μg/ml) and samples were collected at OD_600_ 2.0. Using anti-PrtV polyclonal antiserum, PrtV protein expression and secretion was monitored by immunoblot analysis. Anti-Crp polyclonal antiserum was used to detect Crp, a cytoplasmic protein marker.
